# Smartphones and health promotion

**DOI:** 10.1590/1516-3180.2014.92700716

**Published:** 2014-07-22

**Authors:** Alessandro Wasum Mariani, Paulo Manuel Pêgo-Fernandes

**Affiliations:** I MD. Thoracic Surgeon, Instituto do Coração (InCor), Hospital das Clínicas (HC), Faculdade de Medicina da Universidade de São Paulo (FMUSP), São Paulo, Brazil; II MD, PhD. Titular Professor, Discipline of Thoracic Surgery, Instituto do Coração (InCor), Hospital das Clínicas (HC), Faculdade de Medicina da Universidade de São Paulo (FMUSP), São Paulo, Brazil.

There is no doubt that technological development has made major contributions towards improvement of health worldwide. There are countless examples: diagnostic tools like computed tomography and magnetic resonance imaging, and therapeutic measures like minimally invasive surgery and radiotherapy, among many others. Since both hardware and software are constantly under development, day by day, it can be expected not only that improvements will be made to current devices but also that brand new devices will appear.

In the general field of technology, the market for so-called smartphones is the cutting-edge segment. Use of smartphones or similar devices, like the now-superseded personal digital assistant (PDA) and the recently introduced tablets, as instruments for delivering care is not new. These devices can carry instantly needed information such as pharmaceutical specialty compendiums, emergency books and medical calculators, along with many other medical applications.^1^

However, this year, some of the main technology companies around the world have turned to a new focus of technology usage to promote health through direct use by patients. New devices and special software applications (frequently called apps) are being created for a myriad of health promotion purposes focusing on patients.^2^ One of the first of these "mobile health monitoring device initiatives" was the integration of smartphones with the heart rate monitors frequently used by professional and amateur athletes. In the beginning, there was only one application, which gave heart rate information in real time. However, the new devices go further: they can measure distances through integration with GPS, estimate calorie consumption, record the type and intensity of exercise and, ultimately, organize all these data and present them in a simpler form for interpretation.

Other examples of mobile heath monitoring devices available on the market that have already been integrated with smartphones include personal sleep monitors, blood glucose monitors and blood pressure cuffs.^2^ One shared characteristic among these devices is the possibility of recording the information and creating structured ways to interpret it, like graphics and spreadsheets. Another important characteristic that makes the mix of smartphone and personal health monitoring devices so interesting is their internet capability, which opens up a great range of applications, like warnings that can be sent if blood glucose levels are too low. 

This industry trend seems to be confirmed by some rumors over the internet that one of the major smartphone companies will include a host of health-tracking features on its next generation smartphones, including "the ability to help users track weight, pulse, blood pressure, hydration and blood glucose levels".^3^ In addition, the software could track common parameters like the number of steps taken, quantity of calories burned and distance travelled. These rumors clearly indicate the direction that other companies are likely to follow.

There are few studies measuring the possible impact of these devices on public health. Nonetheless, use of the terms mobile and health monitors has been increasing over the years in the PubMed database. This trend probably demonstrates medical researchers' interest in this emerging field located between technology for consumer use and medical science. One example of this is a meta-analysis on mobile devices and physical activity behavior that has been published. The authors concluded that research using mobile devices is gaining in popularity, and suggested that this platform is an effective means for influencing physical activity behavior.^4^ Another expected impact is better self-control among patients in relation to chronic conditions such as diabetes or hypertension,^5^ guided by mobile heath monitoring devices. However, there are no definitive studies on this subject. 

In conclusion, a flood of new mobile devices directed towards personal healthcare can be expected. In particular, these will integrate existing devices like blood glucose monitors and heart rate monitors with smartphones, using mobile phone internet connection capability to its fullest extent. The real impact of this commercial and technological trend will only become fully understood over the years to come.
